# Editorial: Women in science - rheumatology 2023

**DOI:** 10.3389/fmed.2024.1379276

**Published:** 2024-02-26

**Authors:** Garifallia Sakellariou, Silvia Piantoni

**Affiliations:** ^1^Department of Internal Medicine and Therapeutics, Università di Pavia, Pavia, Italy; ^2^General Medicine Unit, Istituti Clinici Scientifici Maugeri IRCCS Pavia, Pavia, Italy; ^3^Rheumatology and Clinical Immunology Unit, Department of Clinical and Experimental Sciences, Azienda Socio-Sanitaria Territoriale (ASST) Spedali Civili, University of Brescia, Brescia, Italy

**Keywords:** rheumatology, women, gender gap in academic achievement, diversity, research

The number of women involved in the fields of science and technology, including medicine, has relevantly increased in the last decades. However, despite gender parity in the universities is a largely achieved goal, there is still a widening gap as women try to undertake an academic career. Women are even more under-represented when analyzing data from the higher levels of academic organizations, such as scientific academies or science councils. In 2015, an analysis based 69 science academies worldwide highlighted that women represented 10% or less of members (1). In addition to this limited inclusion at high levels, women are required to achieve higher standards to obtain tenured positions and fundings, with often smaller grants compared to men, and still tend to be under-represented among conference speakers, editors and academic positions (2). As diversity is an essential component of innovation, the difficulty in achieving gender balance should be seen as a significant barrier in scientific progress.

Rheumatology, a discipline that has witnessed significant innovations in the last decades and in which the amount of scientific research has relevantly grown, makes no exception. Although women represent half of the rheumatology workforce in several countries (3, 4), there is a lack of diversity in senior positions, and a limited number of women is involved in the highest levels of editorial boards of the relevant scientific journals (5). The presence of women is also limited among invited speakers and moderators at the European Alliance of Associations for Rheumatology (EULAR) congress, accounting for less than half of researchers, varying from 40% to 43%. Although the proportion has increased compared to the past editions of the same event, the gender imbalance is still very relevant (6).

Several associations worldwide are trying to fill this gap, as this aspect still represents a significant issue. For instance, the Association of Women in Rheumatology (AWIR) (7) or the Italian association Reumatologhe Donne (ReDo) (8) advocate the greater involvement of women in all the areas of rheumatology, including the clinical and the research fields.

In 2021, a Research Topic to promote the visibility of female researchers in the area of rheumatology was launched (9), and the same initiative was proposes in 2023, providing an opportunity to increase the dissemination of the results of research led by female scientists. In line with this principle, we included in this Research Topic studies in which women had a leading or senior position, covering various areas.

The article by Richter et al. deals with the topic of technological innovation for proximity medicine, a process that was relevantly accelerated by the COVID-19 pandemics. In this study, the authors performed a nationwide survey in Germany, reaching 205 participants all over the country, to assess the use of telemedicine during this time, including lockdown periods. In this analysis, they demonstrate that despite external constraints and the impossibility to access freely to the rheumatology centers, the use of these technologies was partly limited due to different hurdles, such as inadequate technology or connection, lack of adequate reimbursement, poor evidence on safety or clinical efficacy and lack of demand from patients. This analysis provides insight on the barriers that should be addressed in subsequent research.

Women also lead and participate to relevant and innovative clinical research. In the study by Crotti et al. patients with axial spondyloarthritis not suitable for treatment with biologic disease modifying anti-rheumatic drugs (bDMARDs) received intravenous neridronate. This treatment allowed to achieve a significant clinical and imaging response, as assessed by MRI, within 60 days from treatment. This study suggests therefore a potential treatment option when biological disease modifying anti-rheumatic drugs administration is not possible. In line with this focus on clinical research, Del Giudice et al. review in their paper the various possible off-label applications of treatment with canakinumab in the pediatric setting, encompassing monogenic and multifactorial autoinflammatory diseases, hyperferritinemic syndromes and a variety of rare disorders, for which options with in-label drugs are absent or limited.

The role of women is also relevant in the search for new biomarkers, also in rare diseases. Jonasdottir et al. investigate one of the possible mechanisms determining the increased thrombotic risk seen in ANCA-associated vasculitides. The authors assessed plasma myeloperoxidase-positive extracellular vesicles in AAV patients, whose concentrations were higher in active disease. Their effect was evaluated *in vitro*, assessing the impact on the formation of thrombin, which was increased by the presence of vesicles, thus suggesting a role in determining the increased thrombotic risk.

The articles grouped in this Research Topic display the quality and variety of research work led by women, including clinical and translational research, assessment of new technologies and research on disease mechanisms and biomarkers, allowing the achievement of relevant results. Although the number of women in the field of rheumatology is increasing, representing a relevant proportion of the workforce, there is still a great effort to make to guarantee an equal representation of genders in academia, and, in particular, in senior positions and as research leaders.

Although the EULAR has acknowledged this gap and has identified possible interventions to promote women's advancement in academic rheumatology, there are still several steps that need to be made to achieve this goal (10).

## Author contributions

GS: Writing—original draft, Writing—review & editing. SP: Conceptualization, Writing—original draft, Writing—review & editing.
